# Supplementary figures and images for: Selective enhancement of attentional networks in college table tennis athletes: a preliminary investigation
Source: PeerJ. 2016 Dec 6;4:e2762. doi: 10.7717/peerj.2762 (PMC5144722; doi:10.7717/peerj.2762)

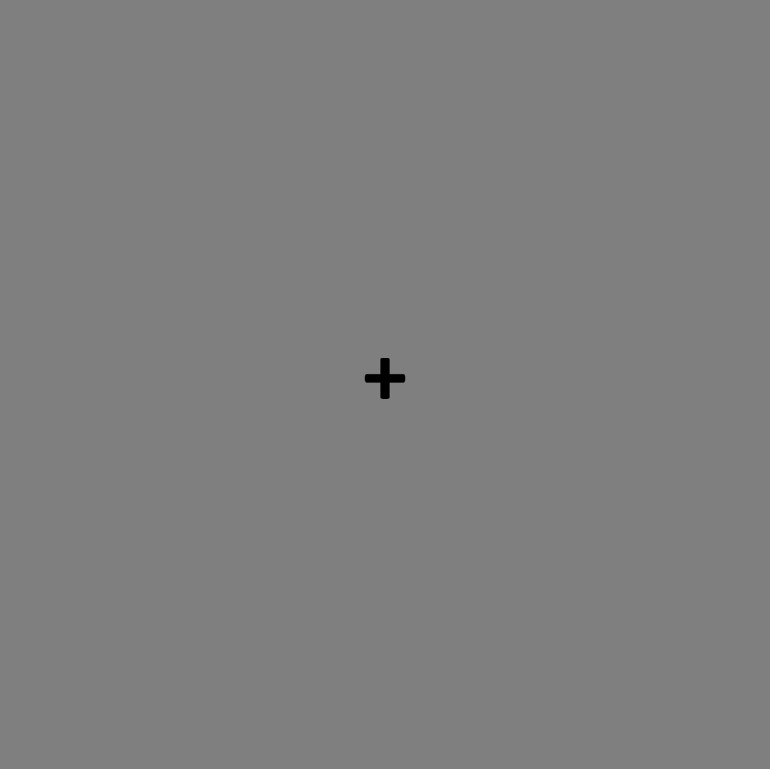

Supplement: Supplemental Information 1 [file peerj-04-2762-s004.zip › program/cue0.jpg]

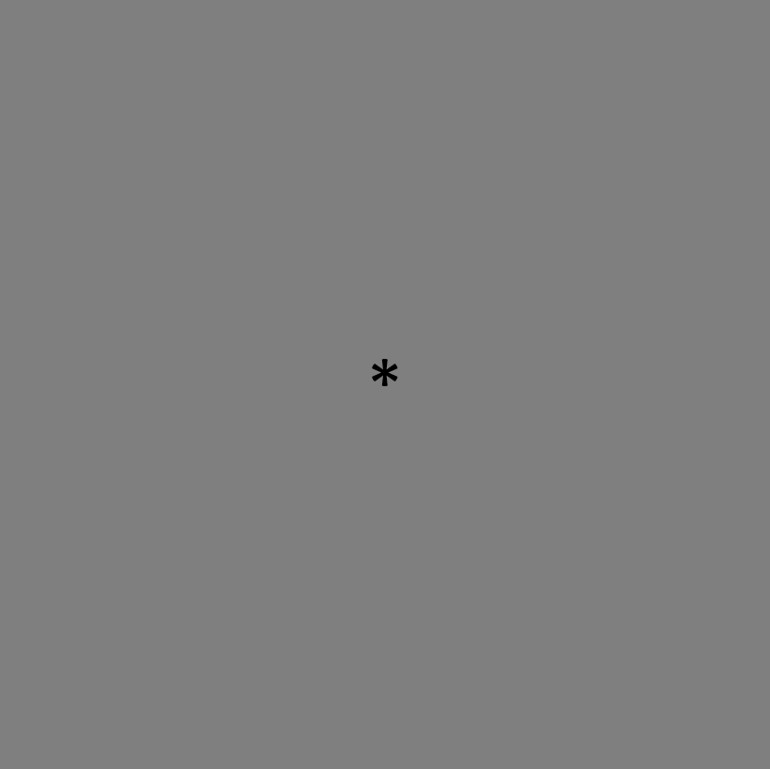

Supplement: Supplemental Information 1 [file peerj-04-2762-s004.zip › program/cue1.jpg]

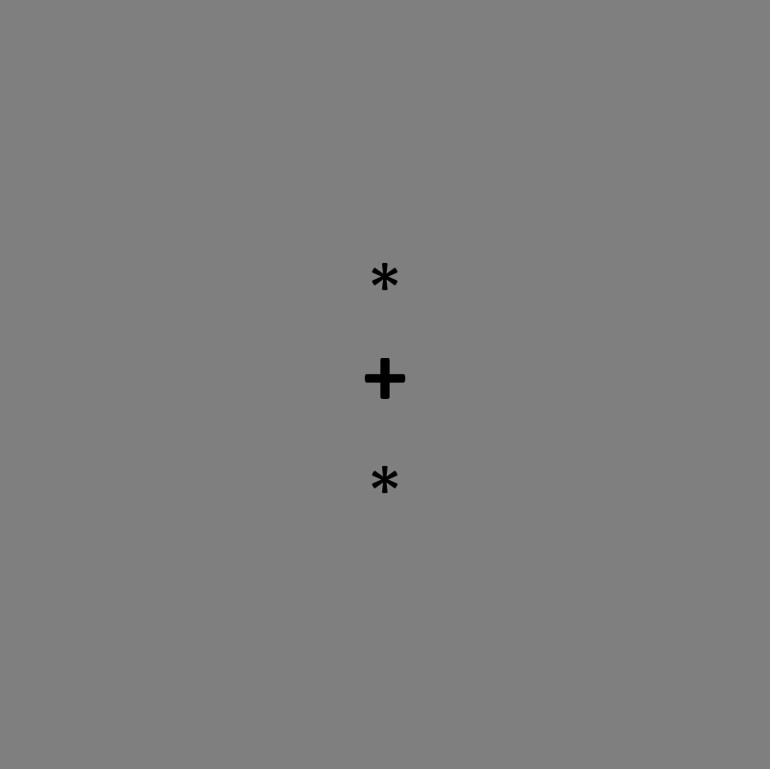

Supplement: Supplemental Information 1 [file peerj-04-2762-s004.zip › program/cue2.jpg]

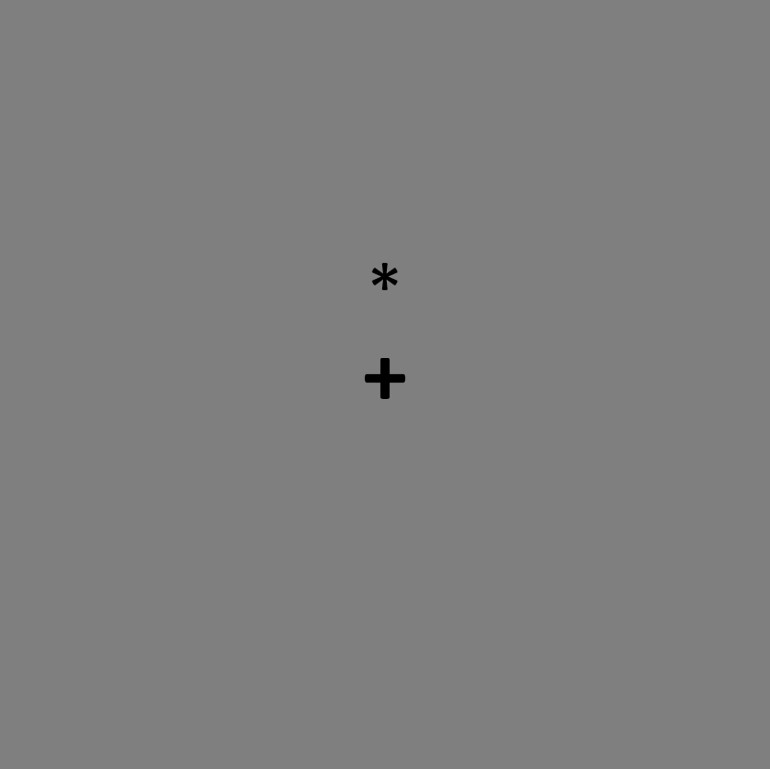

Supplement: Supplemental Information 1 [file peerj-04-2762-s004.zip › program/cue3.jpg]

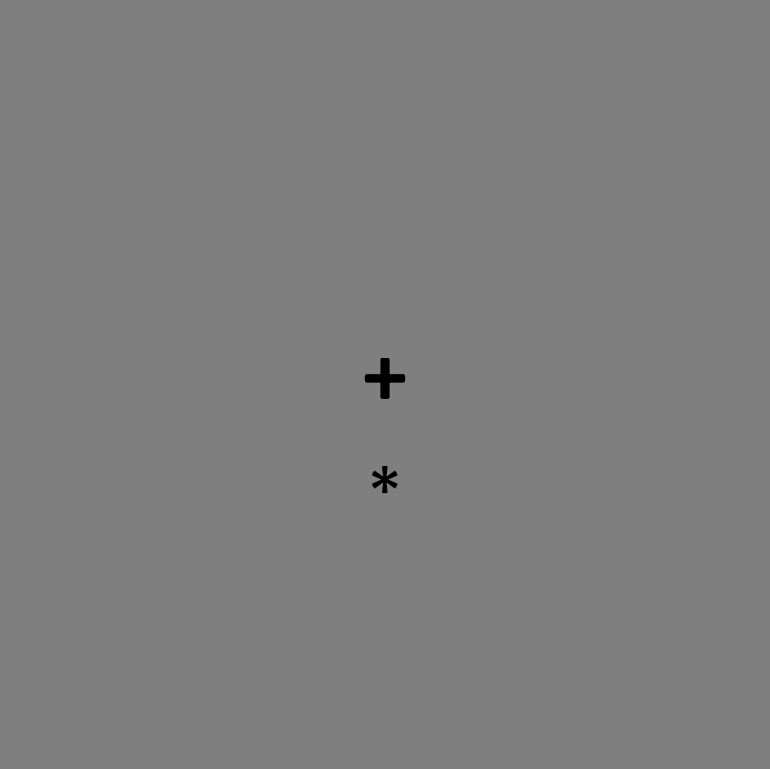

Supplement: Supplemental Information 1 [file peerj-04-2762-s004.zip › program/cue4.jpg]

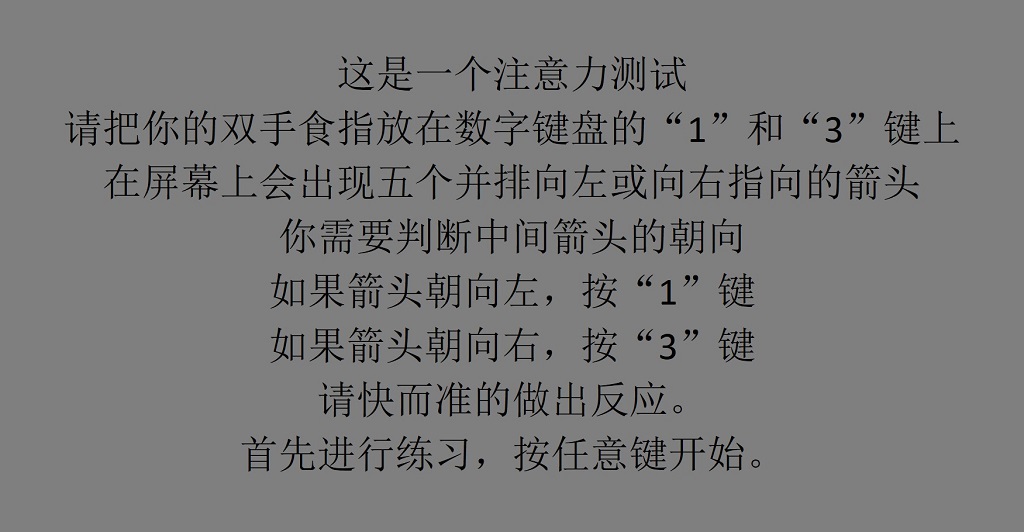

Supplement: Supplemental Information 1 [file peerj-04-2762-s004.zip › program/intro_ANT.jpg]

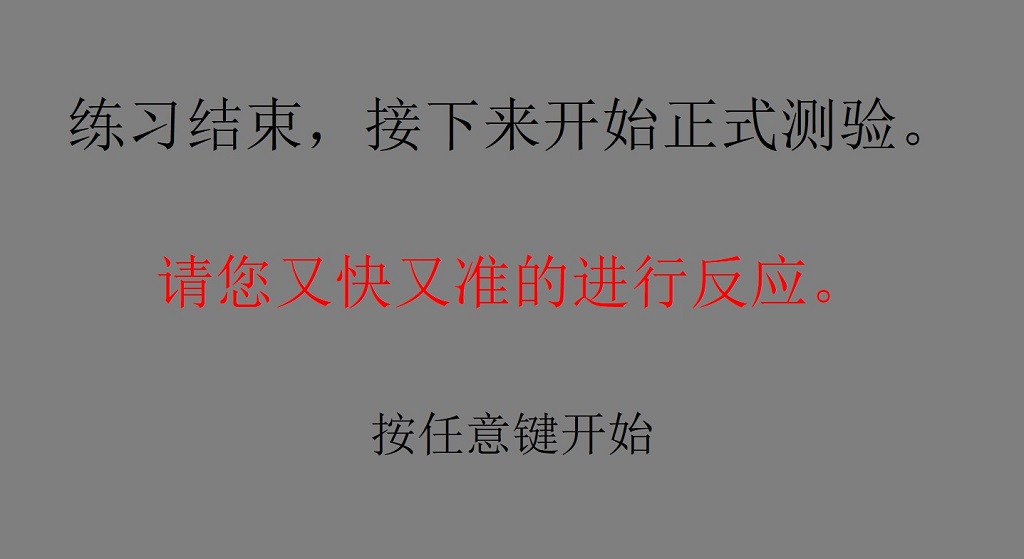

Supplement: Supplemental Information 1 [file peerj-04-2762-s004.zip › program/main_ANT.jpg]

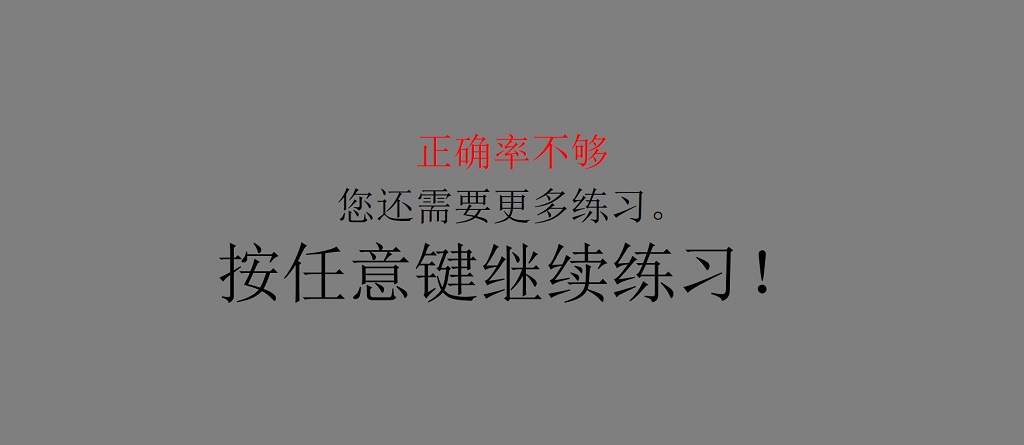

Supplement: Supplemental Information 1 [file peerj-04-2762-s004.zip › program/moreprc_ANT.jpg]

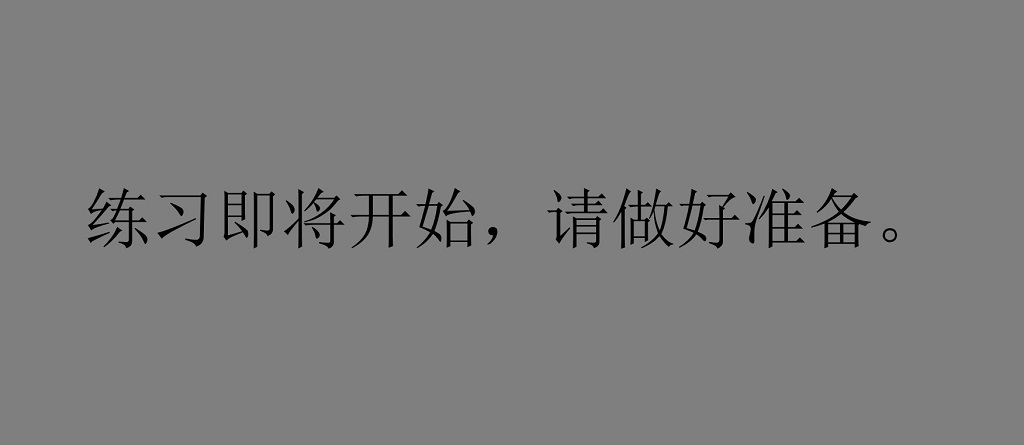

Supplement: Supplemental Information 1 [file peerj-04-2762-s004.zip › program/prc_ANT.jpg]

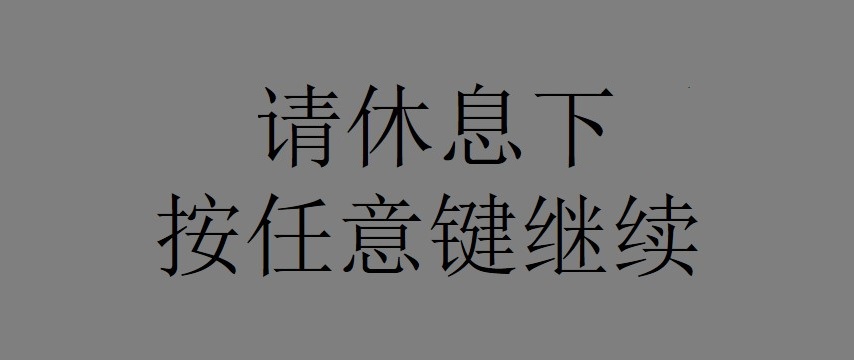

Supplement: Supplemental Information 1 [file peerj-04-2762-s004.zip › program/rest_ANT.jpg]
